# Supplementary material for: Comparison of Gonadotropin-Releasing Hormone versus Estrogen-Based Fixed-Time Artificial Insemination Protocols in Grazing Bos taurus Suckled Beef Cows
Source: Animals (Basel). 2023 Sep 4;13(17):2803. doi: 10.3390/ani13172803 (PMC10487215; doi:10.3390/ani13172803)
Supplement: Supplementary file 1 [file animals-13-02803-s001.zip › animals-2566948-supplementary.pdf]

Table S1. Reproductive outcomes and BCS assessment according to fixed-time artificial insemination (FTAI) protocols.

		Experiment 1[a]				Experiment 2[b]			
**FTAI Protocols**		7-Day Estradiol	7-Day GnRH	7 & 7 Estradiol	7 & 7 GnRH	6-Day Estradiol	7-Day Estradiol	7-Day GnRH	7 & 7 GnRH
**Total cows**	n	59	59	60	57	116	115	116	115
**BCS[1]**		3.70	3.68	3.66	3.71	2.79	2.81	2.80	2.8
**Ovarian status[2]**	Cycling, n (%)	38 (64.4)	38 (64.4)	37 (61.6)	38 (66.6)	58 (50.1)	58 (50.4)	57 (49.1)	59 (51.3)
	No-CL/LF, n (%)	17 (28.8)	17 (28.8)	20 (33.3)	16 (28.1)	33 (28.4)	31 (26.9)	32 (27.5)	30 (26.0)
	No-CL/SF, n (%)	4 (6.7)	4 (6.7)	3 (5.0)	3 (5.2)	25 (21.5)	26 (22.7)	27 (23.2)	26 (22.6)
**Presence of CL[3]**	n (%)	34 (57.6)	54 (91.5)	52 (86.6)	56 (98.2)	--	--	--	--
**Estrus expression[4]**	Yes, n (%)	39 (66.1)	47 (79.7)	45 (75.0)	46 (80.8)	96 (82.8)	97 (84.3)	99 (85.3)	103 (89.6)
	No, n (%)	20 (33.9)	12 (20.3)	15 (25. 0)	11 (19.2)	20 (17.2)	18 (15.7)	17 (14.7)	12 (10.4)
**Pregnant cows[5]**	n (%)	29 (49.1%)	36 (61.0%)	20 (33.3%)	41 (71.9%)	65 (56.0%)	59 (51.3%)	61 (52.5%)	78 (67.8%)
**BCS difference[6]**		-0.9	-0.88	-0.86	-0.89	0.29	0.33	0.33	0.34

[a,b] Within a row and Experiment, values with different superscripts differ ($P < 0.05$).

[a] FTAI protocols: 7-Day Estradiol = cows were administrated 2 mg of estradiol benzoate (EB) + IVPD insert on Day -9 and 1 mg of estradiol cypionate (ECP) + IVPD removal on Day -2, 7-Day GnRH = cows received 10 µg of GnRH + IVPD insert on Day -10, IVPD removal on Day -3 and GnRH at the time of FTAI, 7 & 7 Estradiol = cows were administrated PG + IVPD insert on Day -16, EB on Day -9 and IVPD removal + ECP on Day -2, and 7 & 7 GnRH = cows received PG + IVPD insert on Day -17, GnRH on Day -10, IVPD removal on Day -3

and GnRH at the time of FTAI.

[b] FTAI protocols: 6-Day Estradiol = cows were administrated EB + IVPD insert on Day -9, IVPD removal on Day -3 and GnRH at the time of FTAI. The 7-Day Estradiol, 7-Day GnRH and 7 & 7 GnRH protocols as described for Experiment 1.

[1] Initial BCS assessment at initiation of FTAI protocol (scale 1 to 5 as described by [1]).

[2] Based on ultrasonographic examination before the initiation of FTAI protocols. Cows with a CL were considered to be cycling, cows without a CL and with a follicle ≥ 10 mm were considered no-CL with large follicles (No-CL/LF), and cows without a CL and with follicles < 10 mm were considered no-CL with small follicles (No-CL/SF).

[3] Presence of at least one CL determined by ultrasonography at the time of IVPD insert removal.

[4] Based on tail paint score at the time of FTAI. Yes = ≥50% of paint rubbed off; and No = <50 of paint rubbed off.

[5] Pregnancy status determined by transrectal ultrasonography 35 to 45 d after FTAI.

[6] BCS difference between the 1st assessment (initiation of FTAI protocol) and the 2nd assessment (pregnancy diagnosis).
